# The Discovery of the Role of Outer Membrane Vesicles against Bacteria

**DOI:** 10.3390/biomedicines10102399

**Published:** 2022-09-26

**Authors:** Sofia Combo, Sérgio Mendes, Kaare Magne Nielsen, Gabriela Jorge da Silva, Sara Domingues

**Affiliations:** 1 Faculty of Pharmacy, University of Coimbra, 3000-548 Coimbra, Portugal; 2 Center for Neuroscience and Cell Biology, University of Coimbra, 3004-504 Coimbra, Portugal; 3 Department of Life Sciences and Health, Faculty of Health Sciences at Oslo Metropolitan University, 0130 Oslo, Norway

**Keywords:** outer membrane vesicles, antimicrobial activity, Gram-negative bacteria, Gram-positive bacteria

## Abstract

Gram-negative bacteria are intrinsically resistant to many commercialized antibiotics. The outer membrane (OM) of Gram-negative bacteria prevents the entry of such antibiotics. Outer membrane vesicles (OMV) are naturally released from the OM of Gram-negative bacteria for a range of purposes, including competition with other bacteria. OMV may carry, as part of the membrane or lumen, molecules with antibacterial activity. Such OMV can be exposed to and can fuse with the cell surface of different bacterial species. In this review we consider how OMV can be used as tools to deliver antimicrobial agents. This includes the characteristics of OMV production and how this process can be used to create the desired antibacterial activity of OMV.

## 1. Introduction

Membrane vesicles (MV) are formed and released by a broad range of cells, from bacteria to human cells, with different nomenclature attributed according to the budding cell [1,2,3]. These MV are mostly spheres from the membrane of the cells with a large size range from 20 nm to 10 µm, depending on the donor cell [4,5]. The formation of vesicles occurs by vesiculation from living cells as a result of a disruption of the membrane caused by internal mechanisms or induced by an external signal. Depending on the donor, the bacterial MV have different functions, and the same cell can also produce MV with different functions. The main function of MV is the transport of different molecules, such as lipids, proteins, and nucleic acids. The molecules that constitute the cargo of the MV as well as their target will differ depending on the stimuli [6,7,8,9]. The MV show diverse functions from secretion of toxins and virulence factors to the modulation of the target cell, acquisition of nutrients and even resistance to stress [8,10,11,12].

Depending on their constitution and biogenesis, MV secreted by Gram-negative bacteria can be classified as outer membrane vesicles (OMV), outer-inner membrane vesicles, explosive outer membrane vesicles or tube-shaped membranous structures [13]. The most common MV from Gram-negative bacteria are the OMV, also named membrane blebs or outer membrane blebs [6,14]. MV released by Gram-positive bacteria include the cytoplasmic membrane vesicles and the tube-shaped membranous structures [13].

Antibiotic resistance is due to the bacteria’s ability of adaptation to the action of antimicrobial molecules as well as to prevent them reaching the target site [15,16]. OMV have a natural role in antimicrobial resistance since they can act as a decoy or remove antibiotics that cross the cell wall, allowing bacteria to survive [6,16]. New approaches have been developed to understand whether OMV may be used as a tool to deliver antibiotics against bacteria. Due to the composition similarity of OMV and the outer membrane (OM) of the cell wall of Gram-negative bacteria, delivery of OMV content into Gram-negative bacterial cells is more efficient; nonetheless, an antimicrobial effect can also be seen against Gram-positive bacteria. Additional advantages of OMV as antibiotic delivery tools include their stability, the cargo protection against enzymatic degradation, the ability to incorporate both hydrophilic and hydrophobic molecules and the capacity to selectively target other bacterial cells [17]. Several challenges have been reported including the need to optimize the loading of different antibiotics, as well as the binding of OMV with the desired target cell and resulting toxicity.

Although different types of MV are secreted by bacteria, here we focus on OMV, the archetypal vesicles of Gram-negative bacteria. We discuss key aspects of bacterial OMV and their potential role as a delivery tool of molecules with antibacterial activity. Emphasis will be given to the biogenesis and the antimicrobial activity of OMV. Different studies have shown OMV activity against different bacterial species due to the carriage of antibiotic molecules, or due to some molecules that are naturally present in OMV such as lysins, which behave as an antibacterial molecule when exposed to recipient bacteria [12].

## 2. OMV Biogenesis in Gram-Negative Bacteria

Gram-negative bacteria have an envelope comprising an OM and an inner membrane (IM) with a periplasmic space in between, which contains a layer of peptidoglycan (PG). In the OM there are lipopolysaccharides (LPS) linked covalently by the lipidic moiety and proteins bound as β-barrels, while in the IM the proteins are bound as α-helical [18].

The destabilization of ligations in a bacterium’s cell wall can lead to the detachment of the OM from the cell membrane. Consequently, the natural stabilization of the molecular charges allows the formation of the OMV [18,19]. OMV are nanostructures with size range between 20 and 250 nm that are secreted from the bacteria’s OM, being composed of phospholipids, LPS and outer membrane proteins (OMP) [19]. During formation, molecules such as nucleic acids and proteins from the periplasm and cytoplasm of the cells can be localized to the lumen of the vesicle, making it a vehicle for antibiotic resistance and virulence dissemination [18,20]. Some bacteria can also incorporate external antibiotics into vesicles, allowing isolation of antibiotics inside OMV and cell membrane stabilization [6].

**Figure 1:**
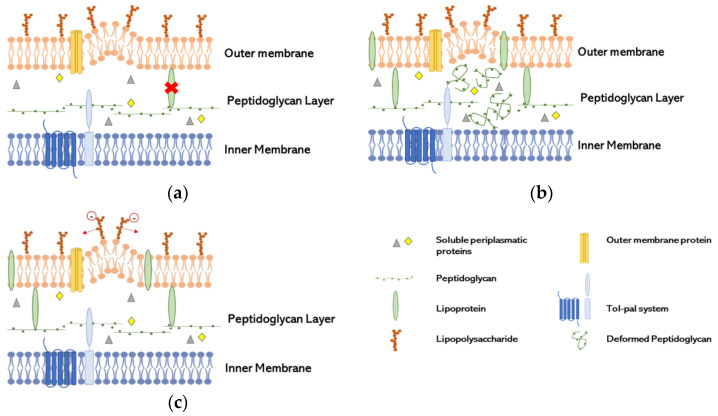
Models of OMV biogenesis (**a**) Vesiculation caused by the absence of lipoprotein links. (**b**) Vesiculation caused by accumulation of misfolded peptidoglycan. (**c**) Vesiculation caused by negatively charged lipopolysaccharide.

The first step to the formation of OMV is the disruption of the connection OM-PG-IM without damage or loss of membrane integrity [18]. To explain the formation of OMV, three models have been created (Figure 1), which are not mutually exclusive: deficiency of lipoprotein (LPP) or its links in OM; increase of PG or other lipids residues; repulsion of negatively charged LPS [19].

1. LPP links deficiency (Figure 1a): the presence of LPP in the unbound form has been found in OMV, indicating that the covalent links were broken, or their distribution was not homogenous, since the conversion of free-form LPP into bound form is reversible [21]. These characteristics seems to be induced by the non-proportional growth of the OM compared with the PG layer [22]. The relation of the lack of link between OmpA and PG has been proven to be essential to the production of OMV in *Salmonella* spp. [23].
2. Increase of misfolded PG (Figure 1b): Autolysins have a role in cleaving the covalent links of PG, resulting in cell wall remodeling. The lack of these enzymes increases the amount of peptides in periplasmatic space and other components leading to turgor pressure and therefore to OMV formation. Several studies explore the lack of autolysins to increase the concentration of proteins in the periplasmatic space and therefore converge to this model [24,25].
3. Repulsion of negatively charged LPS (Figure 1c): A study suggested that the repulsion of negatively charge B-band LPS in cells exposed to gentamicin, with great affinity to LPS, induce the release of OMV as a way of antibiotic resistance in which gentamicin was incorporated into OMV. That repulsion increased the production of vesicles in *P. aeruginosa* [6].

Several molecules are important components and regulators of OMV, including as a regulator of OMV protein composition, such as OmpA from *A. baumannii* [26]. Cells have several mechanisms of response to the disruptions of the connection OM-PG-IM that can be different between species. In *E. coli*, if mutations lead to the absence of LPP, Omp may substitute it [19]. σ^E^, a transcriptional factor and a modulator of OMV formation, is activated to respond to the increase of misfolded OMP and consequently downregulates OmpA and LPP by MicA and Reg26, respectively [18]. Other molecules such as DegP, which behaves as a periplasmatic chaperone at high temperature and a protease at low temperature, seems to prevent the accumulation of proteinaceous waste in periplasmatic space [18]. However, for some perturbations such as LPS with highly charged O-antigen in OM, those molecules are enriched in *P. aeruginosa* OMV. OMV seem to be a bacterium’s own mechanism of defense for the stabilization of the OM charges and therefore cell survival [2].

In general, in the biogenesis of OMV, an increase of the space between the connection OM-PG-IM is necessary. However, its complex formation allows the presence of multiple molecules in OMV that are involved in some essential survival mechanisms of the cell.

To produce higher levels of OMV, growth conditions may be manipulated, including change or addition of parameters such as temperature, antibiotics, serum, active oxygen species mimic molecules, EDTA and lack of amino acids [6,27,28,29,30,31]. The increase of temperature may precipitate proteins increasing the space between OM and IM and leading to the disruption of the LPP links [32,33]. In addition, the use of other physical methods such as electroporation, extrusion and sonication are used to increase the production of OMV, but also to load non-natural molecules inside, once they cause high damage to the cell wall [34,35,36,37,38,39].

## 3. OMV Functions

OMV have several functions that are related with their diverse molecular composition (Table 1), which can be different for each bacterium but also differs with different environmental inducers. Due to its complex composition, one OMV can have more than one function. OMV are used by Gram-negative bacteria to secrete toxins and other virulence factors that can induce cytotoxicity activity, but also other molecules that are important for modulation and invasion of the host that are capable to induce responses from host organisms. In addition, the OMV can also carry molecules responsible for improving the bacterial survival such as those associated with signaling, biofilm production or gene transfer. Beyond the capability to survive, OMV also have an ability to attach to other bacteria, and deliver enzymes known to have antibacterial effect [40].

## 4. Optimization of OMV Production

Several vesiculation-stimulating agents, such as antibiotics and other stimulating factors, such as the presence of serum or limitation of amino acids, can be used to destabilize the cell wall of Gram-negative bacteria and hence increase formation of vesicles [6,27,28,29,30].

Exposure to some antibiotics increases the production of OMV, such as in *P. aeruginosa,* where OMV secretion is enhanced by ciprofloxacin [33,56], or in *Stenotrophomonas maltophilia,* where ciprofloxacin and imipenem increased secretion of OMV but with different compositions and through different mechanisms of formation [57].

Increased temperatures cause the misfolding of proteins and consequently lead to an accumulation of proteins in the periplasmatic space. The accumulation will lead to the formation of OMV through the created pressure [32,33]. However, for some Gram-negative bacteria, OMV formation is regulated by temperature, where low temperature allows vesiculation and a high temperature decreases production or has no effect [58,59]. The effects of temperature changes with the bacterial species may be related with the presence of enzymes with the ability to change their function at higher temperatures, such as heat shock protein DegP (HtrA) in *E. coli* that can change from chaperones to protease [60] or MucD in *P. aeruginosa* that can acquire protease functions [61].

The presence of serum can have some antibacterial effects on bacteria, related with their own components, namely antibodies [31]. However, the hyperproduction of OMV seems to be a resistance mechanism in *Neisseria gonorrhoeae* and *Haemophilus influenzae* [27,28]. Reactive oxygen species mimic molecules, such as hydrogen peroxidase, that have an impact on OMV overproduction in *P. aeruginosa* [59]. The chelating agent ethylenediaminetetra-acetic acid (EDTA) has been proven to be effective in the stimulation of the production of OMV from *Neisseria meningitidis* by capturing the calcium ions from the medium that allows the stabilization of the membrane [62,63]. There are also reports that show that limitation of amino acids induces and regulates the production of OMV in *E. coli* and in *Francisella* spp. [29,30].

## 5. Antimicrobial Activity of OMV

The OMV antimicrobial activity is appealing for treatment purposes, especially against Gram-negative bacteria since it allows bridging with its cell wall. During OMV biogenesis, some molecules with antibacterial activity will be naturally included in the vesicle lumen (Table 1). At the same time, several environmental inductors can enhance or contribute to the inclusion of those molecules into the OMV. In both cases, OMV can act as an antimicrobial agent.

### 5.1. OMV with Natural Antimicrobial Activity Cargo

The functions and roles of autolysin, also known as murein hydrolase [64,65], have being explored in *Bacillus* spp. [66]. Autolysins are usually PG-hydrolyzing endogenous enzymes that naturally lyse the peptide bridges in the PG layer [67], although there are some exceptions where it can have glycosidic activity [68]. There are several types of autolysins related with different mechanisms, such as protein and toxin secretion [69], flagellar formation [70,71], cell separation [72] and antibacterial activity associated to bacterial competition [12,73]. Its relation with vesicles and antimicrobial activity was observed for the first time in *P. aeruginosa* [6]. The presence of autolysins in OMV seem to be related with its normal location at the PG layer, being included in the cargo of the OMV during the bleb of the OM [6,64]. However, a recent study in *Lysobacter* spp. found that the distribution of the L5 enzyme, a bacteriolytic peptidase, may not be random, because it is only present in bacteria when this is exposed to a 30% sucrose medium. This study demonstrated that this autolysin is unevenly placed through the periplasmatic space, specifically where the vesiculation occurred and therefore L5 seems to be a factor in OMV biogenesis [74].

There are a few studies that show the antibacterial activity of vesicles with autolysins, such as peptidoglycan hydrolases, from *P. aeruginosa* PAO1 against different species of Gram-negative bacteria, such as *E. coli* DH5α and *P. aeruginosa* PAO1, and Gram-positive bacteria such as *Brachybacterium conglomeratum* CCM2134, *Bacillus* spp., *Lactococcus lactis* ATCC 7962 and *Staphylococcus aureus* D2C [6,12,75,76]. The antibacterial activity of peptidoglycan hydrolases from OMV from several Gram-negative bacteria have some affinity to some chemotypes of peptidoglycan from target bacteria, such as the A1Υ chemotype, making it more susceptible to cell lysis [66].

Hemolysin is another type of enzyme that is present in OMV from Gram-negative bacteria, such as *P. aeruginosa* and *E. coli* [6,54]. One hemolysin from *P. aeruginosa* has been shown to be responsible for co-regulation of protein secretion but also injection of toxin proteins into other Gram-negative bacteria, including *E. coli* [77], by type VI secretion system (T6SS) [78]. In addition, T6SS has been shown to be incorporated in OMV from P. aeruginosa [8]. The T6SS system allows the bacteria to compete through the delivery of toxins, that are capable of killing other bacteria [78].

Antimicrobial quinolines, 4-hydroxy-2-heptylquinoline (HHQ) and 4-hydroxy-2-nonylquinoline (HNQ), are synthetized by *P. aeruginosa* and incorporated into OMV, which acted as an antimicrobial molecule against *Staphylococcus epidermidis* successfully [55].

Alkaline phosphatase is an enzyme that has been found active in *P. aeruginosa* and in *Myxococcus xanthus* OMV, where the active packaging of this enzyme is suggested [6,44,65]. Alkaline phosphatase from *E. coli* was shown to have antimicrobial activity against Gram-negative bacteria, namely *P. aeruginosa* [79], however the search for a match between OMV and its antibacterial activity has not yet been proven.

Other groups of enzymes, such as phospholipases and proteases, are present in OMV and may have antibacterial effect, however the need for more studies about their presence and action is necessary [6].

### 5.2. OMV with Loaded Antimicrobial Cargo

Several studies have reported the use of OMV as a delivery system to transport antibiotics into Gram-negative bacteria. There are two main ways to incorporate antibiotics into OMV: the passive loading, where the addition of the antibiotics during bacterial growth is enough to produce antibiotic-carrying OMV (aOMV), and the active loading approach, where the antibiotics are forced to enter or coat the OMV or OM of bacteria, so it can be part of the produced OMV.

#### 5.2.1. Passive Loading

Passive loading methods use diffusion by osmotic gradient but only for hydrophobic positively charged small molecules. These molecules may pass through the lipophilic membrane because of their opposite charges and their similar affinity to water [80]. In these methods, only the components of the medium or environmental characteristics are changed to destabilise the membrane and allow the entrance of antibiotics and other molecules [80,81].

Up to now, only antibiotics that have been demonstrated to pass through the cell wall of Gram-negative bacteria have been inserted into the OMV. Addition of gentamicin to *P. aeruginosa* cells showed the production of OMV-carrying gentamicin [6], which had antibacterial effects against both Gram-negative and Gram-positive bacteria species [12]. These gentamicin-carrying OMV showed antibacterial activity against *P. aeruginosa* 8803, which has a permeability-type resistance to gentamicin [12], highlighting the potential of OMV antimicrobial delivery to overcome resistance. More recently, different antibiotics, such as ceftriaxone, amikacin, azithromycin, ampicillin and levofloxacin, were loaded into *A. baumannii* OMV, which showed antibacterial effects against enterotoxigenic *E. coli*, *Klebsiella pneumoniae* and *P. aeruginosa* without toxic activity in mice [82].

Further studies are necessary to determine the capability of other medium components or environmental changes to affect the cell wall in ways that allow the intentional entrance of antimicrobials.

#### 5.2.2. Active Loading

The active loading methods consist of forcing the entrance of the molecules into the vesicles, normally by physically damaging the cells or the vesicles. There are three main methods for active loading: electroporation, sonication, and extrusion, and they are already commonly used to produce vesicles from animal cells such as exosomes.

Electroporation is an electro-physical method that uses electron impulses to rearrange the OM of the cell with the consequent creation of pores [34,83]. This method is typically used to make cell transfections of DNA, RNA and proteins, however its ability to translocate large molecules leads to an instable cell wall and therefore more cell death by lysis and less efficacy [34]. This method has been successfully used to insert small interference RNA into OMV from *E. coli* [35]. Another study showed the possibility to insert any nanoparticles under 10 nm, such as nanoparticles of gold, into OMV from *P. aeruginosa* through this method, a first step to deliver nanoparticles with antibacterial activity through OMV [84].

Sonication uses ultrasound to compress and decompress cells in order to compromise membrane stability, followed by a second sonication to assemble the membrane fragments and allow the incorporation of external molecules [36]. Mild sonication has been used successfully to induce paclitaxel loading into exosomes from macrophages to treat cancer cells and also to load small RNA into extracellular vesicles from different cell lines [37,85]. This method has already been used in *Haemophilus parasuis* to induce OMV production, however the protein content changed when compared to the natural OMV [86]. As far as we know, the use of sonication in bacterial studies is mostly used to induce cell lysis, and for detection of biofilms; studies related to OMV loading with sonication have not yet been performed [86,87,88,89].

Extrusion involves the mixture of cells and antibiotics added to a syringe extruder and then forced to pass through a porous membrane, under controlled temperature. The hydrostatic fluid pressure will disrupt the membrane, by increasing the axial tension, and allow the drug entrance at the same time that vesicles are formed; however, variation of size and zeta potential can occur [9,38]. Despite the yield in loading and forming vesicles with this method is high, the vesicles may not be homogenous and its impact in protein membrane structures is not clear, though higher pressures may lead to protein damage and cell death [38,39]. Extrusion has been proven to be efficient to coat OMV from *E. coli* with gold nanoparticles as well as to load 5-fluorouracil into OMV from *E. coli* [90,91]. It has also been found in *E. coli* that this method disrupts the CusCBA, an efflux pump associated with toxic metals that belongs to the same family related to antibiotic resistance [92]. As far as we know, this methodology has not been used for active loading of antibiotics, however it seems a promising technique to produce OMV with antibacterial activity.

Other methods of active loading include a drastic change of temperature, and utilization of saponin. Severe change of temperature in exosomes has proven to be effective in inducing the incorporation of proteins through the compression and decompression of water molecules in cells that lead to the formation of pores and allow the entrance of molecules into vesicles [36,93]. Incubation with saponin, a surfactant molecule that reacts with cholesterol in cell membranes, has been effective in increasing the loading of molecules into extracellular vesicles without altering the size or even the zeta potential [9]. Although there is no cholesterol in Gram-negative bacteria, an analogue called hopanoid has been discovered [94]. Further studies are necessary to understand the possibility of this method in the production of OMV in Gram-negative bacteria.

## 6. Merging of Vesicles with Target Cells and Delivery of OMV Cargo

The merging of vesicles with Gram-negative target cells occurs in three steps. The first is the membrane contact, followed by the mixing of lipids and fusion of the external layer of the membrane and finally the reorganization of the inner lipidic layer through pore formation and finally mixing of content [95]. The first step requires a high amount of energy that can be given by fusogenic agents. These agents can disrupt the lipidic membrane through the influence of transition from lamellar bilayer-phase lipids into inverted hexagonal-phase lipids; lipid quality is crucial for the membrane rearrangement and content mixing [96,97]. Some of these agents are positive ions, small organic molecules or physical damage to the membrane, such as temperature, in which the difference between them is the energy harnessing process [95].

**Figure 2:**
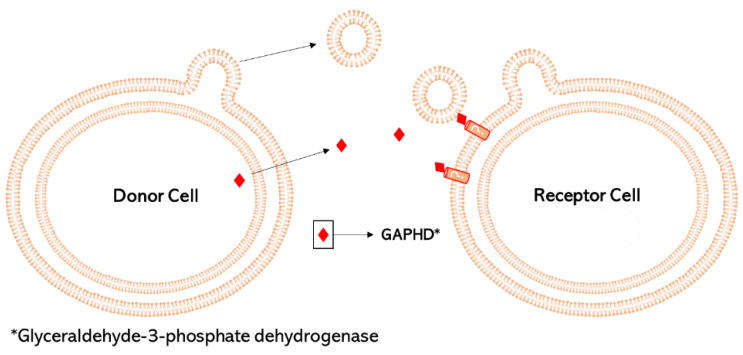
Merging of OMV with bacterial membrane with the glyceraldehyde-3-phosphate dehydrogenase (GAPDH) released by *M. xanthus* as a signal molecule.

Attachment of some vesicles can be explained by the charges of Mg^2+^ and Ca^2+^ at the bacterial membrane. These ions can form salt-bridges between the vesicles and the membrane of exposed cells, that are rich in those ions [12]. Increasing the composition and concentration of ions in the medium reduces the repulsive electrostatic force, allowing particle aggregation. A recent study demonstrated that acidic pH enhances the merging of OMV-OMV from *E. coli* [98]. More recently, a fusogenic enzyme, glyceraldehyde-3-phosphate dehydrogenase (GAPDH), has been discovered alongside OMV from *M. xanthus* and it seems to modulate the fusion with *E. coli* cells (Figure 2) [65].

Vesicles from several Gram-negative bacterial species can promote the lysis of other bacteria, despite the specific interactions related with different PG chemotypes mentioned above [73]. For instance, *P. aeruginosa* vesicles do not merge with the cell wall of *S. aureus*, but rather tend to attach and release their content, such as gentamicin and autolysins, into the extracellular matrix, allowing enzyme activity and therefore antibacterial activity [12]. Although the antibacterial effect is similar to the antibiotic free effect, the antibiotic is delivered near the target, which may increase the success of Gram-positive infection treatments. S-layered species, such as *Aneurinibacillus thermoaerophilus* and *Bacillus* spp., prevent the passage of vesicles through the cell wall but not the attachment of the vesicles from *P. aeruginosa* [69].

In all these cases, the equilibrium of the membrane charges seems to be mandatory. It is necessary that new studies determine if there is a difference between the merging of the different vesicles with other species of bacteria and what cell signaling is associated with the merging after the attachment to the cell wall [65,99,100].

## 7. Other Nano-Sized Techniques Used for Delivery of Antibiotics

Nowadays, new techniques are developed as a different approach to antibiotic delivery, specifically the coating of nanoparticles with OMV membrane and nanotransformation of antibiotics [101,102]. These techniques have been shown to reduce the toxicity associated with OMV [103,104]. A recent study demonstrated that OMV and extracellular vesicles, from *E. coli* and *S. aureus*, respectively, with an antibiotic coat have in vitro and in vivo antibacterial efficacy against *S. aureus*. However, this study only considered the entrance of the vesicles into the infected macrophages and not into the bacteria. Nonetheless, it was proven that it is possible to use the OMV membrane as a coat for antibiotics such as vancomycin and rifampicin with a diameter of approximately 90 nm [104]. A recent study explored the same principle by using extrusion to coat rifampicin-loaded mesoporous silica nanoparticles with OMV from *E. coli*. Unlike the previous study, it was proven the entrance of these vesicles into *E. coli* bacteria but not into *S. aureus* [103].

A novel mechanism of repurposing antibiotics is the sonochemistry that is capable of nanotransforming vancomycin, an antibiotic used against Gram-positive bacteria, to pass through and disrupt the OMs of Gram-negative bacteria. This technique has proven to be effective against *E. coli* and *P. aeruginosa* bacteria [102].

Due to the similarity to the OM composition, natural OMV represent an advantage relative to synthetic nanoparticles, which lack the intercellular interaction’s essential ability to promote trafficking and delivery of antibiotics into bacterial cells [3]. However, the combination of the new approaches is still in its beginning and further developments and applied outcomes are expected.

## 8. Conclusions

OMV from Gram-negative bacteria have innate antibacterial activity due to the incorporation of several enzymes such as lysins. The incorporation of non-natural molecules into OMV with additional antibacterial effect has been shown. This suggests that a delivery system could be developed to overcome the OM barrier of the Gram-negative bacteria and that bacterial OMV can be used to deliver antibiotics to targeted populations of Gram-negative and -positive pathogenic bacteria. The OMV antimicrobial effect will depend on their cargo and the bacterial species targeted, as well as the resistance mechanism present in the target bacterial cells. The repurposing of antibiotics that are ineffective due to the permeability barrier of the cell wall of bacteria may also be possible with the use of OMV.

The industrial production of OMV can be enhanced by changing the growth conditions or through different techniques that force cell damage; an alternative to OMV production with active lumen content is coating of the OMV with nanoparticles. However, several technical challenges that hamper the use of OMV remain, such as the types of MV isolated, the purification yield, and the optimal technique to produce vesicles with desired lumen content, especially hydrophilic molecules which cannot enter by passive loading. The reduction of the LPS toxicity is another point that needs to be optimized in order to reduce the immunogenic potential of OMV; for instance, the engineering of strains with altered LPS [105] might be a solution to produce OMV with reduced cytotoxicity. There is also still the need to better understand the target populations, including the specifics of the interaction between OMV and the target as well as subsequent host cell reactions.

Overall, despite the challenges that must still be overcome, OMV represent a cost-effective and safe drug delivery tool, representing a promising alternative for the treatment of bacterial infections caused by antibiotic-resistant bacteria and for the repurposing of antibiotics that are not usually effective against Gram-negative bacteria.

## Figures and Tables

**Table 1:** Functions of OMV according to their composition.

Function	Species	Active Factor/Molecular Structure	How	Host/Target	Effect	Reference
**Secretion of Toxins and Other Virulence Factors**	*A. baumannii*	Outer membrane protein A (OMPA)	Regulating the induction of cell death in host	Mitochondria and nucleus from host cells	Cytotoxicity activity	[41,42,43]
	*P. aeruginosa*	Hemolytic phospholipase C (Cif virulence factor)	Released directly into cytoplasm	Airway epithelial cells	Cytotoxicity activity	[44]
	*Helicobacter pylori*	Vacuolating toxin (VacA)	OMV enter the gastric mucosa and binds to MKN28 cells	MKN28 cells	Cytotoxicity activity	[2]
	*E. coli*	Shiga toxin 1 and 2	−	−	−	[45]
	*E. coli*	Peptidoglycan-associated LPP	Release of peptidoglycan-associated LPP from OMV, enhanced by ampicillin	−	−	[10]
**Adhesion and Biofilms**	*A. baumannii*	AbFhaB/FhaC system	FhaC protein transports AbFhaB exoprotein to the bacterial surface	*A. baumannii*	−	[46,47]
**Invasion of Host**	*P. aeruginosa*	Small RNA (sRNA)	Attenuation of IL–8 secretion and neutrophil infiltration	Host immune system	Reduction of host innate immune response	[48]
**Modulation**	*A. baumannii*	LPS	Mediation of toll like receptors (TLRs) like TLR4 and TLR2 in macrophages that release chemokines and cytokines to recruit neutrophils	Mice macrophages	Induction of pulmonary inflammatory reaction	[11]
**Mechanism of Resistance**	*Moraxella catarrhalis*	OMV membrane	Trapping azoles even in the presence of other antibiotic that increase its action	Bacteria and fungus like *Candida albicans*	Defence against combined antibiotics	[49]
	*A. baumannii* *Salmonella* Typhi	OMV membrane	Act as a decoy for antibiotics, Polymixin B	−	Protection of bacterial cells	[3,16]
**Gene Transfer**	*E. coli*	*eae*, *stx1* and *stx2*, and *uidA* genes	−	Non-competent *E. coli*	Resistance to β-galactams	[45]
	*K. pneumoniae*	Plasmid with resistant gene to β-lactams	−	*Burkholderia cepacia*, *E. coli*, *P. aeruginosa* and *Salmonella enterica*	Resistance to β-lactams	[50]
	*Salmonella* Typhi	Resistance gene to Polymixin B	−	−	Resistance to Polymixin B, OMV as a decoy in cocultures	[3]
	*E. coli*	*bla*_CTX-M-15_ gene on pESBL plasmid	−	*Enterobacteriaceae*	−	[51]
	*A. baumannii*	*bla*_NDM-1_ gene on plasmid	−	*A. baumannii* and *E. coli*	−	[7]
**Acquisition of Nutrients**	*P. aeruginosa*	T6SS substrate TseF (Type VI secretion system effector for Fe uptake)	Incorporation of T6SS substrate TseF into OMV by reacting with iron binding PQS molecule	−	Iron acquisition	[8]
	*Bordetella pertussis*	Iron receptors and iron binding proteins	−	−	Iron acquisition	[52]
**Signalling**	*Xylella fastidiosa*	Diffuse signalling factor 2 (DSF2)	Regulation of expression of virulence and pathogenic determinants	*Xylella fastidiosa*	Virulence and pathogenicity	[53]
**Bacterial Mortality/Competition**	*P. aeruginosa*	Peptidoglycan hydrolases (autolysins)	−	*P. aeruginosa* resistant to gentamicin	Antibacterial effect	[12]
	*P. aeruginosa* and *E. coli*	Hemolysins	Co-regulation of protein secretion	−	Antibacterial effect	[6,54]
	*P. aeruginosa*	Quinolines	−	*Staphylococcus epidermidis*	Antibacterial effect	[55]
